# Adverse Impacts of Temporomandibular Disorders Symptoms and Tooth Loss on Psychological States and Oral Health-Related Quality of Life During the COVID-19 Pandemic Lockdown

**DOI:** 10.3389/fpubh.2022.899582

**Published:** 2022-07-08

**Authors:** Siwei Weng, Sicong Hou, Xiuping Jiao, Yun Sun

**Affiliations:** ^1^Department of Stomatology, Clinical Traditional Chinese Medicine College of Yangzhou University, Yangzhou, China; ^2^Department of Gastroenterology, Affiliated Hospital of Yangzhou University, Yangzhou University, Yangzhou, China; ^3^Health Management Center, Affiliated Hospital of Yangzhou University, Yangzhou, China

**Keywords:** COVID-19, TMD symptoms, tooth loss, mental health, Oral Health-Related Quality of Life (OHRQoL), lockdown

## Abstract

**Background:**

Emotion and quality of life may have been impacted by the coronavirus disease 2019 (COVID-19) crisis, especially in the lockdown. The impact of temporomandibular disorders (TMD) symptoms and tooth loss on mental status and Oral Health-Related Quality of Life (OHRQoL) are not fully understood in a stressful situation.

**Objectives:**

We aimed to investigate whether TMD and tooth loss were the impaired risks of psychological states and OHRQoL in COVID-19 lockdown, and attempt to explore other potential risk factors.

**Methods:**

This cross-sectional study surveyed residents via an online self-reported questionnaire, when Yangzhou was in lockdown. Demographic data, clinical information, the level of anxiety, depression and OHRQoL were collected and analyzed.

**Results:**

Painful TMD symptoms and tooth loss are the risks of more severe anxiety and depression. TMD symptoms and tooth loss worsened OHRQoL. Lower education degree (OR: 6.31, *P* = 0.019), TMD-related pain symptoms (OR: 10.62, *P* = 0.005), tooth loss (OR: 3.12, *P* = 0.035), sleep disorders (OR: 2.92, *P* = 0.049) and relatively close contacts (OR: 3.95, *P* = 0.020) were verified as risk factors for increased level of anxiety. With respect to depression, low socio-economic status (OR: 6.22, *P* = 0.021), TMD-related pain (OR: 7.35, *P* = 0.012), tooth loss (OR: 4.48, *P* = 0.009), sleep disorders (OR: 5.13, *P* = 0.007) and relatively close contacts (OR: 12.94, *P* = 0.001) were identified as independent factors for developing depression. Additionally, drinking (B: −2.584, *P* = 0.013) and never going to the dental clinic (B: −3.675, *P* = 0.024) were relevant to better OHRQoL, while TMD without pain (B: 2.797, *P* = 0.008), TMD-related pain (B: 12.079, *P* < 0.001), tooth loss (B: 2.546, *P* = 0.006), sleep disorders (B: 2.598, *P* = 0.003) were independent factors for impaired OHRQoL.

**Conclusion:**

Painful TMD symptoms, tooth loss and sleep disorders were the impaired risks of psychological states. TMD symptoms and tooth loss damaged OHRQoL when the city was in lockdown. Therefore, individualized psychological counseling is supposed to maintain control of mental health and OHRQoL under the stressful event.

## Introduction

Temporomandibular disorders (TMD) are a group of conditions that cause pain and dysfunction of the masticatory muscles, temporomandibular joint (TMJ), and the surrounding musculoskeletal structures (1). Being the third most prevalent social dental disease, TMD affects about 10–15% population across the world and the incidence is still rising (2, 3). Typical symptoms of TMD include local pain, limited mandibular movement, and TMJ sounds during movement (4). The etiology of TMD still remains elusive, accumulating evidence suggests that factors from biologic, environmental, social, emotional, and cognitive aspects all play an indispensable role in the occurrence and development of TMD (5). Among these triggers, the contribution of psychological issues involved in emergency or threatening situations like the ones faced with the coronavirus disease 2019 (COVID-19) pandemic, to the onset and duration of TMD has been acknowledged by more and more researchers (6, 7). Psychological and socio-demographic factors, including somatization, depression, stress, anxiety, daytime sleepiness, optimism, gender and age, aggravated pain intensity and pain-related disability in patients with TMD (8). In turn, previous studies showed that TMD patients exhibited higher level of psychological disorders such as anxiety and depression compared with healthy controls (9–11). Patients with self-reported TMD-pain reported higher scores for anxiety, depression, and somatic symptoms compared to patients with no TMD-Pain complaint (12). In addition, ample evidence demonstrates that tooth loss is negatively associated with depression, anxiety and Oral Health-Related Quality of Life (OHRQoL) (13, 14), which is in line with the observation that effective treatment of tooth loss have positive effects on quality of life and mental status (15). Collectively, the influence of the bidirectional relationship between psychosocial and physical factors deserves further clinical attention.

Since the end of 2019, a novel, threatening pandemic, the COVID-19 caused by severe acute respiratory syndrome coronavirus 2(SARS-CoV-2) infection, caused a health crisis worldwide (16–18). Many countries adopted a policy of lockdown aiming to decelerate the contagion, which subsequently made dramatical changes of the daily lifestyle of the residents, and negatively influenced the mental health of individuals (19, 20). Recent studies implicated that outbreak of COVID-19 was supposed to cause emotional disorders, cognition dysfunction, behavioral disturbance and impaired quality of life, especially in population with psychosomatic diseases such as coronary heart disease, irritable bowel disease and psoriasis, in whom psychological distress and somatic diseases could mutually influence one another (21–24). Regarding dentistry, most of the studies indicated adverse effects of COVID-19 on psycho-emotional status (stress, anxiety, depression), which lead to the intensification of TMD symptoms and increased orofacial pain (OFP) (25). However, few studies have examined whether some dental problems may be the risk factors of the mental health and OHRQoL during COVID-19 lockdown.

Dental clinic is confronted with potential risk of transmitting COVID-19. On one hand, multiple dental procedures using ultrasound instruments tend to produce aerosol fog of saliva or blood droplets in the workplace, which has been proven to be a potent carrier of the virus (26); on the other hand, the execution of dental treatment involves close contact between dentist and patients, which further exacerbates the possibility of human-to-human transmission (27, 28). Thus, according to the American Dental Association (ADA) recommendation, all dental treatments, in addition to the emergency treatment, should be delayed.

There was a COVID-19 outbreak in Yangzhou, a tourist city in Eastern China. It was a resurgence of the coronavirus since Wuhan lockdown. Before this resurgence, the pandemic of COVID-19 in China had been at stable status. To cope with it, the government took strict lockdown measures. This cross-sectional study was performed using an online questionnaire to explore potential risk factors of the altered psychological status and OHRQoL during Yangzhou lockdown, thereby modifying the variables for which intervention is feasible, and optimizing the management of susceptible people. We assume that TMD or tooth loss are the risk factors of higher levels of anxiety and depression as well as poorer OHRQoL.

## Methods

### Participants

Due to the impossibility of clinical evaluation because of the social isolation, the evaluation of TMD symptoms, OHRQoL, anxiety, and depression was carried out through online questionnaires. The questionnaires were edited through the Wen-Juan-Xing online platform. Then, an electronic two-dimensional code of the questionnaire was created. When Yangzhou was in the lockdown, every residential district has its own WeChat group. A lot of information about epidemic lockdown was sent through the WeChat group, and the questionnaires were randomly distributed to those groups by the group advocates. All the subjects were residents in these enclosed districts, and the sample size collected during the opening hour had reached the expectation. A total of 300 online questionnaires were randomly distributed, but 23 individuals were excluded from the analysis, including 9 were diagnosed with psychological diseases at the time of our study; 10 had other physical disorders closely related to their psychological state, and the remaining 4 cases did not provide sufficient information. The inclusion criteria were as follows: (1) those who were residents in these enclosed districts during Yangzhou lockdown. (2) volunteered to participate in research. The exclusion criteria included (1) those who did not answer the questionnaire in the opening hours; (2) those who were diagnosed with psychological diseases at the time of our study; (3) those who had other physical disorders closely related to their psychological state. According to Peduzzi et al. (29) and Concato et al. (30), the sample size satisfied the following analysis. This survey was conducted 1 month after the implement of the lockdown. All protocols in the study were carried out in accordance with the principles of the Declaration of Helsinki. This study was approved by the Institutional Review Board and Ethics Committee of Clinical Traditional Chinese Medicine College of Yangzhou University (REC ref 2021-29). We obtained their authorization by signing the informed consent.

### Data Collection

The first part of the questionnaire collects basic information of the participants, including gender, age, occupation, education, economic level, marital status, etc. The second part is some issues related to the epidemic, for example: Were you close contacts of any confirmed cases or familiar with someone who is confirmed case? Have you been vaccinated? Did you join in the voluntary service against the epidemic in the local community?

The third part mainly assesses the presence of symptoms related to TMD. On the basis of the Diagnostic Criteria for Temporomandibular Disorders (DC/TMD), which was translated and validated into Chinese with objective questions about TMD symptoms. The answered questionnaires were analyzed by a specialist in the field and the participants were classed into three groups according to their symptoms: without TMD symptoms, with non-painful TMD symptoms, or painful TMD symptoms (31). In this part, there are also some issues about sleep disorder, tinnitus, tooth loss and so on.

The fourth part focuses on the psychological status and OHRQoL of the responders. The Generalized Anxiety Disorder Scale 7 (GAD-7), which assesses the patient's anxiety over the past month, consists of seven questions, each with four choices, with scores ranging from 0 to 3, for a total of 21 points. The higher the score is, the worse the anxiety is. A score of 0–4 is classified as normal, 5–9 as mild anxiety, 10–14 as moderate anxiety and 15–21 as severe anxiety (32). Depression was assessed with Patient Health Questionnaire-9 (PHQ-9). There are nine questions, each score is 0–3 points, with a total score of 27 points. Zero to four is minimal depression, 5–9 mild, 10–14 moderate, 15–19 moderately severe and 20~27 severe (33). To evaluate the OHRQoL of the participants, the Oral Health Impact Profile-14 (OHIP-14) scale was applied. It covers 14 items in seven areas with five choices in each item. The score is 0 to 4 for each item with a total score of 56, which is inversely proportional to the level of OHRQoL (34).

### Statistical Analysis

Statistical analysis was performed using SPSS version 22.0 (IBM SPSS Statistics, USA). Continuous variables were presented as mean ± standard deviation (SD) and categorical variables were presented as proportions. Univariate logistic regression was performed to identify potential modifiers of psychological stress.

In univariate analysis, the screening criteria for candidate predictors was *P* < 0.05. Then, multivariate analysis was further used to eliminate the variables with no statistical significance. *P* < 0.05 in multivariate analysis was considered statistically significant. Odds ratios (OR) were applied to measure the correlation between variables and outcomes of psychological stress.

Using the added scores of OHIP-14 as a continuous outcome, multiple linear regression analyses were performed adjusted for all variables. The coefficients 95% confidence intervals (CI) for Beta and *P*-values were calculated.

## Result

A total of 300 online questionnaires were randomly distributed among the lockdown population, 23 individuals were excluded as described above. Basic information is shown in Table 1. The age is mainly distributed in the 30–40 stage, and the male: female ratio was 103:174. Seventy- seven percent were married, 17.33% single, and 6.14% divorced. In regard to the occupation, 28.88% worked in state -owned units; 31.41% in private units; 23.47% worked freelance; and the remaining 16.25% had retired. In terms of education level, 78.34% got a bachelor's degree or higher. Smokers and drinkers accounted for 11.91% and 20.58%, respectively.

**Table 1:** Demographic characteristics of patients included in the study.

**Variables**	**Mean ±SD or *n* (%)**
Gender, *n* (%)	
Male	103 (37.18%)
Female	174 (62.82%)
Age, years (mean ± SD)	
20–30 30–40 40–50 50–60 60–70	37 (13.36%) 110 (39.71%) 41 (14.80%) 57 (20.58%) 32(11.55%)
Marital status, *n* (%)	
Married	212 (76.53%)
Single	48 (17.33%)
Divorced	17 (6.14%)
Occupation, *n* (%)	
State-owned units	80 (28.88%)
Private units	87 (31.41%)
Freelance work	65 (23.47%)
Retired	45 (16.25%)
Education level, *n* (%)	
Elementary	4 (1.44%)
Middle school	56 (20.22%)
Bachelor or above	217 (78.34%)
Socio-economic status, *n* (%)	
Very low	28 (10.11%)
Low	84 (30.32)
Middle	79 (28.52%)
High	86 (31.05%)
Smoking	33 (11.91%)
Drinking	57 (20.58%)
Vaccination	232 (83.75%)
Voluntary service	119 (42.96%)
Relatively close contacts	123 (44.40%)
Frequency of going to the dental clinics, *n* (%)	
Regular check	28 (10.11%)
Seldom	34 (12.27%)
When necessary	188 (67.87%)
Never	27 (9.75%)
Sleep disorders	145 (52.35%)
Tinnitus	97 (35.02%)
Tooth loss	87 (31.41%)
TMD symptoms, *n* (%)	
No symptoms of TMD	206 (74.37%)
Symptoms of TMD without pain	51 (18.41%)
Symptoms of TMD with pain	20 (7.22%)

Concerning the TMD symptoms, most participates (74.37%) did not report any symptoms of TMD, 51 patients suffered from TMD without pain, while the rest complained of TMD with pain. A total of 145 patients (52.35%) reported sleep disorders. Tinnitus and tooth loss were present in 35.02% and 31.41% patients, respectively. When talking about the frequency of going to the dental clinics, 10.11% of the participates had a regular check, while the majority turned to a dental clinic when necessary. After the outbreak of the epidemic, 119 patients in our study joined in the voluntary service against the epidemic in the local community, and most of the population (83.75%) accepted vaccine injection. In addition, 123 (44.40%) were considered as relatively close contacts.

When using 5 as the cut-off value for the GAD-7 scale, the incidence of anxiety was 46.21% (128/277), with 86 in the mild group, 23 in the moderate group and 19 in the severe group (Table 2). In terms of depression, 117 (42.24%) scored ≥5 for the PHQ-9 scale, regarded as the existence of depression, and among these, 82 (29.60%) presented mild depression, 19 (6.86%) moderate, and 16 (5.78%) moderately severe. During the closure, the mean score of the OHRQoL assessed by OHIP-14 is 11.22 ± 7.66.

**Table 2:** Level of anxiety, depression and OHRQoL among patients included in the study.

**Variables**	***n* (%)**	**Mean ±SD**
Anxiety (GAD-7)		
No	149 (53.79%)	1.96 ± 1.39
Mild	86 (31.05%)	7.01 ± 1.29
Moderate	23 (8.30%)	11.35 ± 1.20
Severe	19 (6.86%)	15.42 ± 1.43
Depression (PHQ-9)		
No	160 (57.76%)	1.99 ± 1.26
Mild	82 (29.60%)	5.99 ± 1.09
Moderate	19 (6.86%)	11.87 ± 1.07
Moderately severe	16 (5.78%)	16.63 ± 1.49
Severe	0	/
OHRQoL(OHIP-14)		11.22 ± 7.66

To explore the influencing factors of anxiety in the situation of COVID-19 lockdown, we first applied univariate logistics regression to screen potential variables. As shown in Table 3, compared with retired, working in state-owned [OR: 0.30 (95% CI: 0.10–0.88), *P* = 0.028) or private units [OR: 0.33 (95% CI: 0.11–0.95), *P* = 0.040] tended to have lower anxiety. Lower education degree [OR: 5.45 (95% CI: 2.34–12.72), *P* < 0.001], poorer socio-economic status, regular check at the dental clinics, [OR: 17.85 (95% CI: 1.73–184.56), *P* = 0.016], TMD-related pain [OR: 14.46 (95% CI: 3.89–44.79), *P* < 0.001], tooth loss [OR: 2.12 (95% CI: 1.02–4.44), *P* = 0.044], sleep disorders [OR: 3.10 (95% CI: 1.18–8.19), *P* = 0.022], tinnitus [OR: 5.53 (95% CI: 2.49–12.51), *P* < 0.001] and relatively close contacts [OR: 4.58 (95% CI: 1.74–12.05), *P* = 0.002] were significantly related to higher anxiety. After adjustment by multivariant analysis, lower education degree [OR: 6.31 (95% CI: 1.35–29.37), *P* = 0.019], TMD-related pain [OR: 10.62 (95% CI: 2.07–54.59), *P* = 0.005), tooth loss [OR: 3.12 (95% CI: 1.08–8.99), *P* = 0.035], sleep disorders [OR: 2.92 (95% CI: 1.003–15.30), *P* = 0.049) and relatively close contacts [OR: 3.95 (95% CI: 1.24–12.60), *P* = 0.020] were verified as risk factors for more severe anxiety.

**Table 3:** Factors associated with elevated anxiety.

**Variables**	**Unitivariable analysis**			**Multivariable analysis**		
	***P***	**OR**	**95%CI**	***P***	**OR**	**95%CI**
Gender (male)	0.196	0.58	0.25–1.33			
Age						
20–30	0.153	0.17	0.01–1.94			
30–40	0.258	0.47	0.13–1.74			
40–50	0.906	0.91	0.21–4.02			
50–60	0.875	0.89	0.22–3.61			
60–70						
Marital status						
Married	0.356	0.54	0.15–1.99			
Single	0.230	0.35	0.06–1.94			
Divorced						
Occupation						
State-owned units	0.028*	0.30	0.10–0.88	0.288	2.96	0.40–21.85
Private units	0.040*	0.33	0.11–0.95	0.408	2.22	0.34–14.57
Freelance work	0.473	0.67	0.22–2.02			
Retired						
Education level						
Elementary 0	0.201	5.45	0.41–73.18			
middle School	<0.001*	5.45	2.34–12.72	0.019*	6.31	1.35–29.37
Bachelor or above						
Socio-economic status						
Very low	<0.001*	22.89	4.66–112.51	0.054	11.37	0.96–135.37
Low	0.025*	3.15	1.15–8.61	0.078	3.33	0.87–12.76
Middle	0.028*	3.07	1.13–8.35	0.052	3.53	0.99–12.60
High						
Smoking	0.531	0.64	0.16–2.62			
Drinking	0.482	0.70	0.27–1.87			
Frequency of going to the dental clinics						
Regular check	0.016*	17.85	1.73–184.56	0.443	3.22	0.17–64.46
Seldom	0.082	8.08	0.77–84.69			
When necessary	0.073	6.83	0.84–55.81			
Never						
Voluntary service	0.603	0.82	0.39–1.71			
Vaccination	0.744	1.17	0.46–2.97			
TMD symptoms						
No symptoms of TMD						
Symptoms of TMD without pain	0.676	0.81	0.30–2.16			
Symptoms of TMD with pain	<0.001*	14.46	3.89–44.79	0.005*	10.62	2.07–54.59
Tooth loss	0.044*	2.12	1.02–4.44	0.035*	3.12	1.08–8.99
Sleep disorder	0.022*	3.10	1.18–8.19	0.049*	2.92	1.003–15.30
Tinnitus	<0.001*	5.53	2.49–12.51	0.314	1.76	0.59–5.27
Relatively close contacts	0.002*	4.58	1.74–12.05	0.020*	3.95	1.24–12.60

* *, P < 0.05 was considered significant*.

With respect to depression, after using univariate logistics regression to screen potential variables, we performed multivariant analysis. Low socio-economic status [OR: 6.22 (95% CI: 1.33–29.14), *P* =0.021], TMD-related pain [OR: 7.35 (95% CI: 1.55–34.81), *P* = 0.012], tooth loss [OR: 4.48 (95% CI: 1.45–13.80), *P* = 0.009], sleep disorders (OR: 5.13 (95% CI: 1.55–16.98), *P* = 0.007] and relatively close contacts [OR: 12.94 (95% CI: 2.98–56.15), *P* = 0.001] were identified to be independent factors for progression of depression (Table 4).

**Table 4:** Factors associated with elevated depression.

**Variables**	**Unitivariable analysis**			**Multivariable analysis**		
	***P***	**OR**	**95%CI**	***P***	**OR**	**95%CI**
Gender (male)	0.450	0.71	0.29–1.74			
Age						
20–30	0.451	0.37	0.03–5.00			
30–40	0.169	0.39	0.10–1.49			
40–50	0.807	1.22	0.25–6.07			
50–60	0.411	0.56	0.14–2.25			
60–70						
Marital status						
Married	0.703	1.27	0.37–4.41			
Single	0.547	0.55	0.08–3.87			
Divorced						
Occupation						
State-owned units	0.068	0.36	0.12–1.08			
Private units	0.107	0.41	0.14–1.21			
Freelance work	0.397	0.60	0.18–1.95			
Retired						
Education level						
Elementary	0.183	5.89	0.43–80.32			
Middle school	0.002*	3.91	1.65–9.25	0.192	2.24	0.67–7.49
Bachelor or above						
Socio-economic status						
Very low	0.003*	7.89	1.99–31.28	0.411	2.29	0.32–16.56
Low	0.030*	3.48	1.13–10.77	0.021*	6.22	1.33–29.14
Middle	0.081	2.72	0.88–8.34			
High						
Smoking	0.369	0.533	0.14–2.10			
Drinking	0.803	0.87	0.30–2.51			
Frequency of going to the dental clinics						
Regular check	0.259	3.96	0.36–43.29			
Seldom	0.820	0.71	0.04–13.24			
When necessary	0.233	3.77	0.43–33.38			
Never						
Voluntary service	0.308	0.66	0.29–1.46			
Vaccination	0.420	1.52	0.55–4.19			
TMD symptoms						
No symptoms of TMD						
Symptoms of TMD without pain	0.359	1.74	0.53–5.70			
Symptoms of TMD with pain	<0.001*	15.01	4.48–50.25	0.012*	7.35	1.55–34.81
Tooth loss	0.042*	2.26	1.03–4.97	0.009*	4.48	1.45–13.80
Sleep disorder	0.005*	4.03	1.52–10.73	0.007*	5.13	1.55–16.98
Tinnitus	0.005*	3.35	1.44–7.79	0.205	2.16	0.66–7.06
Relatively close contacts	<0.001*	11.21	3.19–39.49	0.001*	12.94	2.98–56.15

* *, P < 0.05 was considered significant*.

In addition, drinking (B: −2.584, *P* = 0.013) and never going to the dental clinic (B: −3.675, *P* = 0.024) were relevant to better oral related quality of life, while TMD without pain (B: 2.797, *P* = 0.008), TMD-related pain (B: 12.079, *P* < 0.001), tooth loss (B: 2.546, *P* = 0.006), sleep disorders (B: 2.598, *P* = 0.003) were independent risk factors for impaired OHRQoL (Table 5).

**Table 5:** Factors associated with OHRQoL.

**Variable**	**B**	**Std. error**	**Beta (standardized)**	**t**	***P***	**95%CI for B**
Age						
20–30						
30–40	0.242	1.844	0.015	0.131	0.896	−3.390–3.873
40–50	−0.277	2.144	−0.013	−0.129	0.897	−4.500–3.945
50–60	0.571	2.070	0.030	0.276	0.783	3.506–4.648
60–70	−1.076	2.382	−0.045	−0.452	0.652	−5.768–3.616
Gender						
Male						
Female	0.102	0.852	0.006	0.120	0.904	−1.575–1.780
Occupation						
State-owned units	0.418	0.599	0.057	0.698	0.486	−0.762–1.599
Private units	−0.100	0.974	−0.006	−0.103	0.918	−2.018–1.818
Freelance work	0.102	1.435	0.006	0.071	0.943	−2.725–2.929
Retired						
Education level						
Elementary	−0.035	1.675	−0.002	−0.021	0.983	−3.334–3.263
Middle school	1.203	1.898	0.063	0.634	0.527	−2.536–4.941
Bachelor or above						
Marital status						
Married						
Single	0.877	1.318	0.043	0.665	0.506	−1.719–3.473
Divorced	2.576	1.772	0.081	1.454	0.147	−0.914–6.066
Smoking	1.129	1.311	0.048	0.861	0.390	−1.454–3.712
Drinking	−2.584	1.034	−0.136	−2.498	0.013*	−4.620–(−0.547)
Socio-economic status						
Very low	0.385	0.588	0.050	0.654	0.514	−0.774–1.544
Low	−1.084	1.171	−0.065	−0.925	0.356	−3.391–1.224
Middle	1.214	1.072	0.072	1.132	0.259	−0.899–3.326
High						
Frequency of going to the dental clinics						
Regular check	0.946	0.688	0.093	1.376	0.170	−0.408–2.301
Seldom	−0.462	1.322	−0.020	−0.349	0.727	−3.066–2.142
When necessary						
Never	−3.675	1.616	−0.142	−2.275	0.024*	−6.857–(−0.493)
Voluntary service	−0.933	0.924	−0.060	−1.010	0.314	−2.753–0.887
Vaccination	0.004	1.106	0.000	0.004	0.997	−2.174–2.183
TMD symptoms						
No symptoms of TMD						
Symptoms of TMD without pain	2.797	1.051	0.142	2.660	0.008*	0.726–4.868
Symptoms of TMD with pain	12.079	1.732	0.408	6.976	<0.001*	8.669–15.490
Tooth loss	2.546	0.918	0.154	2.773	0.006*	0.738–4.355
Sleep disorder	2.598	0.881	0.169	2.949	0.003*	0.863–4.333
Tinnitus	1.261	0.985	0.078	1.279	0.202	−0.680–3.201
Relatively close contacts	0.168	0.832	0.011	0.202	0.840	−1.470–1.806

* *, P < 0.05 was considered significant*.

In order to assess the impact of these variables to the occurrence of anxiety and depression, logistic regression analyses were performed. The multivariant analysis showed that sleep disorder (OR: 3.468, *P* < 0.001) and relatively close contacts (OR: 6.859, *P* < 0.001) were independent risk factors for the occurrence of anxiety, and 30–40 years old (OR: 6.073, *P* = 0.009), 50–60 years old (OR: 7.857, *P* = 0.005), tooth loss (OR: 1.958, *P* = 0.036), divorced status (OR: 6.155, *P* = 0.012), tinnitus (OR: 2.083, *P* = 0.027) and relatively close contacts (OR: 3.182, *P* < 0.001) were verified as risk factors for increasing the occurrence of depression. Male (OR: 0.541, *P* = 0.049) decreased the occurrence of depression (Tables 6, 7).

**Table 6:** Factors of the occurrence of anxiety.

	**Unitivariable analysis**			**Multivariable analysis**		
	***P***	**OR**	**95%CI**	***P***	**OR**	**95%CI**
Gender (male)	0.033*	0.582	0.354–0.957	0.064	0.546	0.288–1.036
Age						
20–30						
30–40	<0.001*	4.859	2.038–11.588	0.061	3.738	0.94–14.869
40–50	0.025*	3.131	1.158–8.465	0.169	2.997	0.627–14.322
50–60	0.009*	3.5	1.368–8.954	0.187	2.659	0.622–11.367
60–70	0.366	1.648	0.558–4.863			
Marital status						
Married						
Single	0.027*	0.472	0.242–0.920	0.71	0.825	0.299–2.278
Divorced	0.758	1.168	0.434–3.143			
Occupation						
State-owned units						
Private units	0.4	0.77	0.419–1.415			
Freelance work	0.093	0.565	0.291–1.1			
Retired	0.532	0.792	0.381–1.646			
Education level						
Elementary 0						
Middle school	0.945	0.931	0.122–7.081			
Bachelor or above	0.862	0.839	0.116–6.065			
Socio-economic status						
Very low						
Low	0.184	1.875	0.742–4.737			
Middle	0.097	2.202	0.868–5.59			
High	0.019*	3.013	1.197–7.585	0.057	3.623	0.964–13.625
Smoking	0.118	0.543	0.252–1.168			
Drinking	0.486	0.811	0.45–1.461			
Frequency of going to the dental clinics						
Regular check						
Seldom	0.746	0.843	0.3–2.372			
When necessary	0.369	1.45	0.645–3.261			
Never	0.351	1.664	0.571–4.853			
Voluntary service	0.464	1.195	0.742–1.926			
Vaccination	0.172	0.639	0.336–1.214			
TMD symptoms						
No symptoms of TMD						
Symptoms of TMD without pain	0.015*	2.16	1.158–4.03	0.191	1.701	0.768–3.767
Symptoms of TMD with pain	0.002*	6.049	1.953–18.736	0.245	2.314	0.563–9.51
Tooth loss	0.023*	1.812	1.085–3.025	0.469	0.777	0.393–1.537
Sleep disorder	<0.001*	4.957	2.964–8.29	<0.001*	3.468	1.83–6.574
Tinnitus	0.002*	2.188	1.323–3.617	0.944	1.026	0.504–2.088
Relatively close contacts	<0.001*	6.198	3.671–10.464	<0.001*	6.859	3.585–13.121

* *, P < 0.05 was considered significant*.

**Table 7:** Factors of the occurrence of depression.

	**Unitivariable analysis**			**Multivariable analysis**		
	***P***	**OR**	**95% CI**	***P***	**OR**	**95% CI**
Gender (male)	0.009*	0.505	0.303–0.841	0.049*	0.541	0.293–0.999
Age						
20–30						
30–40	<0.001*	8.873	2.945–26.735	0.009*	6.073	1.559–23.662
40–50	0.052	3.414	0.991–11.758			
50–60	<0.001*	12.196	3.805–39.091	0.005*	7.857	1.848–33.415
60–70	0.043*	3.75	1.044–13.472	0.376	2.035	0.422–9.807
Marital status						
Married						
Single	0.011*	0.388	0.188–0.801	0.219	0.547	0.29–1.43
Divorced	0.006*	6.087	1.699–21.809	0.012*	6.155	1.503–25.208
Occupation						
State-owned units						
Private units	0.748	0.904	0.49–1.668			
Freelance work	0.174	0.625	0.318–1.23			
Retired	0.676	1.169	0.562–2.43			
Education level						
Elementary 0						
Middle school	1.000	1.000	0.131–7.605			
Bachelor or above	0.691	0.669	0.093–4.841			
Socio-economic status						
Very low						
Low	0.575	0.78	0.327–1.861			
Middle	0.895	1.061	0.444–2.532			
High	0.818	1.106	0.468–2.616			
Smoking	0.982	1.009	0.483–2.105			
Drinking	0.222	0.685	0.374–1.256			
Frequency of going to the dental clinics						
Regular check						
Seldom	0.245	0.545	0.196–1.515			
When necessary	0.562	0.790	0.357–1.75			
Never	0.127	0.421	0.139–1.277			
Voluntary service	0.359	1.253	0.774–2.027			
Vaccination	0.05*	0.526	0.276–1.001	0.126	0.543	0.249–1.188
TMD symptoms						
No symptoms of TMD						
Symptoms of TMD without pain	0.075	0.548	0.282–1.062			
Symptoms of TMD with pain	0.068	2.441	0.935–6.372			
Tooth loss	<0.001*	2.662	1.581–4.48	0.036*	1.958	1.046–3.664
Sleep disorder	0.002*	2.157	1.324–3.516	0.901	1.041	0.557–1.945
Tinnitus	<0.001*	3.499	2.088–5.864	0.027*	2.083	1.089–3.984
Relatively close contacts	<0.001*	3.42	2.076–5.634	<0.001*	3.182	1.754–5.77

* *, P < 0.05 was considered significant*.

## Discussion

Our findings showed that during the city-wide lockdown caused by SARS-CoV-2, TMD and tooth loss significantly altered psychological stress and OHRQoL. When confronting the stress of COVID-19, people had to change their daily life and behavior, particularly social relationships, which can affect health in varieties of aspects, including mental and physical risks (35). In the field of dentistry, previous studies showed that the pandemic's influence on psychological factors may increase risks of developing TMD symptoms and certain oral diseases, such as bruxism and OFP (25, 36, 37). For instance, a study conducted during the pandemic in Israeli and Polish indicated that COVID-19-related mental disorders led to the intensification of bruxism and TMD symptoms, and consequently OFP increased (38). Gender differences has been proven to be an influential factor contributed to such processes: the anxiety and stress caused by COVID-19 participate in deteriorating stomatognathic diseases, and women were generally more influential than men (36, 39). The cross-sectional study from Wuhan lockdown showed a significantly higher impact of COVID-related stress in TMD patients in comparison to orthodontic patients and control population (40). A prospective study in Italy revealed that as compare with acute/subacute TMD patients, chronic TMD patients were more likely to suffer from increased facial pain severity and COVID-19-related stress (41). However, whether dental problems such as TMD and tooth loss may aggravate psychological stress and deteriorate OHRQoL during COVID-19 lockdown in China was rarely reported before. We attempted to identify potential risk factors to predict psychological disorders and the impaired sense of wellbeing under the stressful event. The results provide data supports to improve the manner of treatments and optimize management for people with dental problems. Additionally, our findings are complementary and reinforce the influence of the bidirectional relationship between psychosocial and physical factors in dentistry.

According to our questionnaire-based analysis, our findings demonstrated that TMD-related pain was significantly correlated with increased psychological stress. Of the 71 subjects who reported TMD symptoms, 20 (28.19%) suffered from painful TMD symptoms, which was found to be responsible for the development of more severe anxiety and depression. There were previous studies showing that chronic pain increases the severity of anxiety, as well as depression (42, 43). Although we didn't investigate the specific reason behind elevated stress, one important factor that could not be ignored is the intense confinement under the context of lockdown, which may exacerbate feelings of loneliness. Therefore, we can infer that “home isolation” could be considered as a critic trigger, which was previously confirmed in another study (44).

Our study revealed that TMD symptoms significantly decreased OHRQoL of the volunteers, suggesting that perception of happiness was impaired by TMD symptoms. Our findings could partially explain why non-steroidal anti-inflammatory drugs (NSAIDs) and selective serotonin reuptake inhibitors (SSRIs) could effectively improve the OHRQoL of TMD patients (45). Thus, it is reasonable to speculate that NSAIDs and SSRIs may be considered to relieve the decline of OHRQoL during the pandemic, especially in those with prominent TMD symptoms.

We discovered that the score of anxiety, depression and oral quality in the group with tooth loss were remarkably higher than those without tooth loss. This phenomenon was in consistence with other investigations, but might be more serious under that situation, which could be, at least in part, attributed to the closure of the dental clinics (46, 47).

Lower economic status and educational level were also identified as important risk factors for predicting the severity of psychological disorders during the lockdown. This finding was in accordance with previous studies, which have linked long-term stress including financial or educational problems to the increased stress (43, 48). One possible explanation for this phenomenon is the limited understanding of the SARS-CoV-2, which can be considered as a direct trigger for psychological stress.

It was widely acknowledged that sleep problem is a crucial factor in emotional burden. As an important health indicator, sleep quality may help strengthen the immune system against infection and facilitate in the regulation of behavior and emotion (49). Previous studies demonstrated that global stressor events (e.g., global pandemic, wars) were critical factors for sleep and psychological disorders (50). In this study, we also verified the negative impact of sleep disorders on psychological distress and OHRQoL, indicating that sleep quality could be a potent target for the management of dental patients during lockdown. Of note, people who never go dental have lower oral quality scores than other groups. This sounds strange but is actually reasonable, because those who never go dental might be quite satisfied with their oral health, which was reported in previous literatures (51, 52).

In the present study, no individuals were diagnosed with COVID-19, but 123 (44.40%) were considered as relatively close contacts and strongly associated with anxiety and depression. Under that situation, people were under the almost complete lockdown and all non-emergency medical and dental treatments were suspended. Most public places such as shops, restaurants were closes. Personal contact with family members and/or friends who are not cohabiting in the same household is prohibited. For the confirmed cases and their closest contacts, medical isolation was conducted, which means separation in isolation wards, nuresd by medical staff. Importantly, the relatively close contacts who contacted of any confirmed cases relatively closely or was familiar with confirmed case, faced much higher risk and more fear of being contaminated by the virus than the general population. Based on this, it is not surprising that relatively close contacts presented higher levels of anxiety and depression. Our findings suggest the importance of timely psychological counseling for reducing the psychological pressure of the relatively close contacts. It's encouraging that online intervention for TMD or psychological problems can be useful at least at its initial stage in times of isolations when face-to-face consultation is prohibited during COVID-19 (25). Further investigation is obviously needed to confirm the role of online intervention during COVID-19 lockdown in more detail.

Our findings should be considered with caution given some limitations of the study. Firstly, the relatively small sample size may have influenced results. Secondly, this study was conducted in one center in one country, which reduces the external validity and the generalizability of the results. In addition, Since, the questionnaires were self-reported, subjectivity is a factor that cannot be ignored when translating these findings into clinical practice. In some cases, the extent of anxiety/depression may be overstated or underestimated, which lead to the severity of the illness may be inaccurate. Hence, these factors should be taken into consideration when using the results of our study. Despite these limitations, it is worth noting that the current study for the first time to investigate the impacts of TMD symptoms and tooth loss on the COVID-19 lockdown related anxiety, depression and OHRQoL.

The results remind us that as oral health professionals, our work shouldn't be limited to just the oral cavity, but the whole person. When treating patients with TMD or tooth loss, additional attention should be paid to their psychological status, so as to support their oral health as well as the integral ability to perform activities of daily living. Further prospective longitudinal studies are necessary to validate our findings, thus extending the clinical application of the current study.

## Conclusion

This study highlights COVD-19 pandemic, especially the lockdown followed have significant adverse impacts on psychological states and OHRQoL in people with TMD symptoms. Those with TMD-related pain, tooth loss and sleep disorders were more prone to experience worse psychological stress. Individualized management such as psychological counseling should be promoted to maintain control of mental health and OHRQoL under the stressful event.

## Data Availability Statement

The original contributions presented in the study are included in the article/supplementary material, further inquiries can be directed to the corresponding authors.

## Ethics Statement

This study was approved by the Institutional Review Board and Ethics Committee of Clinical Traditional Chinese Medicine College of Yangzhou University (REC ref 2021-29). The patients/participants provided their written informed consent to participate in this study. Written informed consent was obtained from the individual(s) for the publication of any potentially identifiable images or data included in this article.

## Author Contributions

SW, SH, XJ, and YS carried out the studies, participated in the experimental design and statistical analysis, and drafted the manuscript. All authors read and approved the final manuscript.

## Funding

This work was supported in part by grant from National Natural Science Foundation of China (No. 31800675 to SH, the Postdoctoral Science Foundation in Jiangsu Province (No. 2018K263C to SH), and 2021 Yangzhou Policy Guidance Plan (Soft Science Research) Project (No.YZ2021231 to XJ).

## Conflict of Interest

The authors declare that the research was conducted in the absence of any commercial or financial relationships that could be construed as a potential conflict of interest.

## Publisher's Note

All claims expressed in this article are solely those of the authors and do not necessarily represent those of their affiliated organizations, or those of the publisher, the editors and the reviewers. Any product that may be evaluated in this article, or claim that may be made by its manufacturer, is not guaranteed or endorsed by the publisher.
